# Optimization of environmental DNA-based methods: a case study for detecting brook trout (*Salvelinus fontinalis*)

**DOI:** 10.7717/peerj.20347

**Published:** 2026-02-11

**Authors:** Erika Myler, Yoamel Milián-García, Tzitziki Loeza-Quintana, Danielle Bourque, Robert H. Hanner

**Affiliations:** 1 Department of Integrative Biology, University of Guelph, Guelph, Ontario, Canada; 2 Ecological and Regulatory (Ecoreg) Solutions Inc., Guelph, Ontario, Canada; 3 SLR Consulting (Canada) Ltd., Guelph, Ontario, Canada

**Keywords:** Environmental DNA, eDNA, eDNA extraction, DNA isolation, qPCR, ddPCR, Metabarcoding

## Abstract

The utility of eDNA for fish species and community monitoring is well-established using targeted amplification (*i.e*., qPCR and ddPCR) and sequencing approaches (*i.e*., metabarcoding). However, the lack of optimized and standardized methods across the eDNA workflow reduces the sensitivity of eDNA surveys and precludes the reliable comparison of findings across studies, respectively. DNA extraction is a prime target for optimization efforts because the extraction method is highly variable across eDNA studies despite being one of the most influential factors in detection efficiency across the entire post-collection workflow. Sequence analysis is arguably the least standardized step in the workflow, with new bioinformatics pipelines frequently emerging in the literature and being implemented with innumerable unique combinations of parameter values. The current study aimed to support the optimization and standardization of eDNA methods for fish detection by assessing two commercial DNA extraction kits. The kits, manufactured by Qiagen and Macherey-Nagel, were evaluated based on cost, time, and performance specifications and the success of brook trout detection by metabarcoding across three bioinformatics pipelines, qPCR, and ddPCR. Our protocols were effective in detecting brook trout in all 20 samples analyzed. Brook trout eDNA was detected by ddPCR in nine (90%) Qiagen extracts but only seven (70%) Macherey-Nagel extracts. The concentration of target DNA determined by ddPCR was significantly greater in Qiagen extracts. In comparison, detection success was equal across the two extraction kits using qPCR (70%) and metabarcoding (100% across all three bioinformatics pipelines). The concentration of target DNA determined by qPCR was not significantly different between Qiagen extracts and Macherey-Nagel extracts; however, the number of target DNA reads determined by metabarcoding was significantly greater in Qiagen extracts using MetaWorks, but no significant difference was found using the MiFish Pipeline. Under our experimental conditions, the Qiagen kit was selected as the preferred kit; while slightly more time-intensive, performance was equal or superior across all analysis methods at a substantially lower cost than the Macherey-Nagel kit. We present this method optimization as a case study which can be applied as a framework for eDNA practitioners to facilitate the evaluation of novel eDNA extraction kits as they become available, against established methods in the field.

## Introduction

Environmental DNA (eDNA) has gained increasing attention and acceptance over the past decade as a highly sensitive, cost-effective, and non-invasive biomonitoring tool for both aquatic and terrestrial ecosystems (Andruszkiewicz et al., 2017; van der Heyde, Bunce & Nevill, 2022; Wang et al., 2021). DNA is shed by all organisms into the environment through various natural processes (*e.g*., excretion, cell sloughing, gamete release) which provides an avenue for species detection from DNA in environmental samples, such as water and soil, without direct organism capture or sighting as is required for conventional surveys (Andruszkiewicz et al., 2017; Rees et al., 2014; van der Heyde, Bunce & Nevill, 2022). It is well-established that eDNA-based detection sensitivity is superior to conventional methods for fishes, which are the dominant taxa of interest in the eDNA literature (Takahashi et al., 2023). Several studies have demonstrated the ability of eDNA-based methods to corroborate conventional survey data and further detect species not captured by conventional methods (Li et al., 2019; McDevitt et al., 2019; Nolan et al., 2023).

The eDNA workflow begins with collecting and extracting eDNA from an environmental substrate followed by targeted (*i.e*., single or few species) and/or community eDNA detection. Quantitative polymerase chain reaction (qPCR, also called real-time PCR) is a targeted detection method that employs species-specific oligonucleotides to detect a single species of interest as DNA is amplified above a set detection threshold. Using fluorescent oligonucleotide probes, DNA is quantified in real-time as it is amplified by PCR. In some cases, droplet digital PCR (ddPCR) is used for targeted detection instead of qPCR due to its higher sensitivity (Doi et al., 2015; Nathan et al., 2014) by fractionating each PCR reaction into thousands of separate droplets. This fractionation allows for absolute quantification, reduces the effect of inhibitory substances, and increases sensitivity, as each droplet per sample is independently scored as a positive or negative amplification.

In contrast to targeted-species detection by qPCR or ddPCR, DNA metabarcoding is capable of multi-taxa identification from complex samples using high-throughput sequencing (HTS) and subsequent bioinformatics analysis of sequence data before matching to a sequence database for taxonomic identification (Taberlet et al., 2012). The use of eDNA metabarcoding and the number of available bioinformatics pipelines for analyzing raw sequence reads is rising (Hakimzadeh et al., 2023). The lack of standardization in sequence analysis methods in the metabarcoding workflow warrants investigating several pipelines to compare their performance on a single dataset. These comparisons are valuable for validating the results and reducing the risk of false negatives or positives arising from a single pipeline’s limitations (Milián-García et al., 2021, 2023).

The need for standardization is apparent from the wide variability in methods reported in the eDNA literature across all stages of the workflow, from study design and sample collection to molecular analysis and interpretation. Best practice guidelines have been recently developed in some jurisdictions (*e.g*., Australia and New Zealand (De Brauwer et al., 2023)) to improve the quality and comparability of eDNA studies. In Canada, the focus of our study, standards for eDNA surveys were recently developed by the Canadian Standards Association (Canadian Standards Association, 2021, 2023), which apply to some aspects of the eDNA workflow and subsequent reporting, though significant gaps remain. These standards were developed with the overarching goals of promoting confidence in eDNA approaches and facilitating comparability across eDNA studies and practitioners (Canadian Standards Association, 2021, 2023). The former presents terminology and minimum reporting standards which span the entire eDNA workflow, from survey design to molecular analysis and interpretation (Canadian Standards Association, 2021). The latter specifically details performance criteria for eDNA analysis using targeted probe-based qPCR (Canadian Standards Association, 2023). At present, Canadian performance standards do not exist for other eDNA analysis methods, including ddPCR and metabarcoding, nor for eDNA sample collection, eDNA extraction, or subsequent processing prior to molecular analysis.

In order to improve the viability of eDNA as a species detection method, the efficacy of methods implemented prior to analysis must be considered. At the forefront of these considerations is DNA extraction. The method by which DNA is extracted from an environmental sample has one of the strongest effects on downstream detection, considering all stages of sample processing following collection (Piggott, 2016; Sanches & Schreier, 2020). There is no effective eDNA-based identification without efficient DNA isolation, regardless of the performance of the downstream molecular or bioinformatic analysis. Studies comparing DNA extraction methods have demonstrated that the choice of method significantly impacts the amount of DNA recovered (Sanches & Schreier, 2020), the detection rate of the target species (Deiner et al., 2015; Guthrie et al., 2023), and the determination of community structure (Djurhuus et al., 2017). The low relative abundance of DNA in environmental samples makes efficient and reliable DNA isolation critical for sensitive eDNA-based detection (Hinlo et al., 2017), as failure to isolate a sufficiently high quantity of DNA will produce a false negative result. Therefore, DNA extraction methods should be optimized to avoid underestimating species presence; however, optimization must consider site- and study-specific factors that influence the performance of a given method (Hinlo et al., 2017). The optimal method depends on a broad range of factors affecting the quantity and quality of eDNA in an environmental sample, including but not limited to sample type, sample collection method, taxa of interest, season, degradation rate, and the concentration of inhibitory substances (Deiner et al., 2018; Hinlo et al., 2017). The efficacy of several extraction methods at the forefront of the eDNA literature has been explored through direct comparison studies (*e.g*., Adrian-Kalchhauser & Burkhardt-Holm, 2016; Deiner et al., 2015; Jeunen et al., 2019; Sanches & Schreier, 2020). While a wide range of commercial DNA extraction kits as well as non-commercial chemical extraction methods have been applied in the eDNA literature, the DNeasy® Blood & Tissue kit (Qiagen, Hilden, Germany) consistently demonstrates superior performance in these comparison studies and was used in just over 50% of published eDNA studies (*n* = 260) despite not being a specialized kit for eDNA extraction or extraction from filters (Wang et al., 2021). Consequently, multiple modifications are needed before it is adopted to isolate genetic material from environmental samples, particularly filters resulting from water filtration (Milián-García, 2022). Authors using this kit for eDNA studies frequently report modifications/optimizations made to the manufacturer’s protocol on a laboratory or project basis (García-Machado et al., 2023; Goldberg et al., 2011; Spens et al., 2017). Recently, Macherey-Nagel (Germany) released the NucleoSpin® eDNA Water kit, developed explicitly for isolating eDNA from water samples after filtration, including inhibitor removal during isolation. Both of these characteristics posit the Macherey-Nagel kit as a promising eDNA extraction kit, which may rival the performance of the Qiagen kit and warrants a comparison study.

In the current study, we compare two commercial DNA extraction kits for the extraction of eDNA captured on filters: 1. DNeasy® Blood & Tissue kit (hereafter, BT kit; Qiagen, Hilden, Germany), and 2. NucleoSpin® eDNA Water kit (hereafter, NW kit; Macherey-Nagel, Düren, Germany). We evaluate the efficacy of each kit for isolating brook trout (*Salvelinus fontinalis*) eDNA from water samples collected at two creeks in Southern Ontario, Canada, where brook trout populations are routinely monitored. The optimal extraction kit is determined based on cost per sample, time of extraction, and success of detection of brook trout eDNA. As the kits’ efficiency in isolating the eDNA of the target species is wholly linked to the analytical methods used for detecting the species, the performance of the kits is assessed using each of the most common DNA-based detection methods used in molecular laboratories for species identification: targeted detection by probe-based qPCR and ddPCR, and community-based detection by metabarcoding. Further, three bioinformatics pipelines for analyzing metabarcoding raw sequence reads to the point of taxonomic assignment are assessed to determine the level of congruence across these methods concerning brook trout detection. Five sequence databases are used for taxonomic assignment with two different classifiers: the basic local alignment search tool (BLAST) and the Ribosomal Database Project (RDP) classifier. We present this work as a case study to guide eDNA practitioners through the process of method optimization on a small sample set to align with study-specific objectives and limitations. We generate preliminary data to support the estimation of effect size in similar contexts to inform study design, particularly sample size optimization.

## Materials and Methods

Portions of this text were previously published as a preprint (Myler et al., 2024).

**Figure 1:**
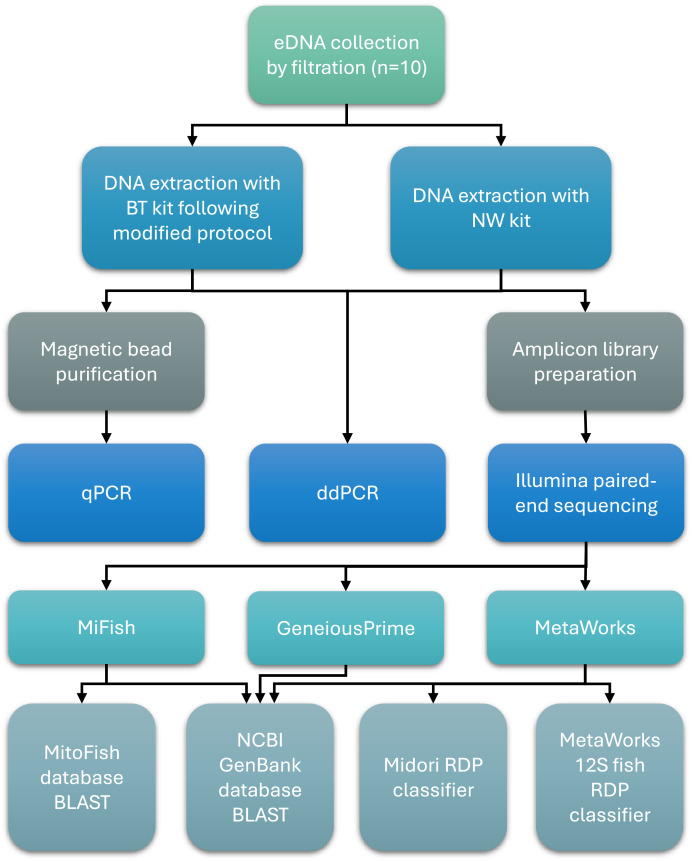
Workflow of sample collection, laboratory processing, and bioinformatics analysis steps.

An overview of key methods for sample collection, laboratory processing, and bioinformatics analysis is presented in Fig. 1.

### Assessment of cost per sample and time of extraction

The cost per sample was calculated using the largest kit size available for purchase for both the BT and NW kits. The manufacturer handbooks for each kit were obtained to identify any additional consumable supplies required for the extraction that are not included in the kit (*e.g*., centrifuge tubes) and must be purchased separately.

To assess the time of extraction, the laboratory protocols for both kits were obtained from their respective manufacturers and reviewed for the following three components: (1) incubation time; (2) centrifugation time; and (3) the number of pipetting steps. A modified protocol optimized in-house for filter samples was used for the BT kit (Milián-García, 2022). Incubation and centrifugation time sum to the total hands-off time, while the number of pipetting steps is linearly correlated with hands-on time. Hands-on time was not determined directly to reduce bias, as this time varies with the number of samples, the experience level of the laboratory personnel, and familiarity with the extraction kit.

### Sample collection and processing

Freshwater (lotic) samples were collected from two creeks in Dufferin County, Ontario, Canada, in July 2020 by on-site filtration using the OSMOS eDNA sampler (Halltech) and mixed cellulose ester filters (MCE; 90% nitrocellulose, 10% cellulose acetate; 5.0 μm pore size; 47 mm diameter) (Advantec MFS, Inc., Dublin, CA, USA). For confidentiality purposes, the names and locations of the two creeks cannot be disclosed. A total of ten spatially explicit zones were sampled; six were sampled from one creek (Creek 1, samples 1–6) and four from the second creek (Creek 2, samples 7–10). Three biological replicates were collected at each zone, and a target volume of 5 L was filtered per sample. Filters were preserved in 95% ethanol in 5 mL centrifuge tubes on-site and stored at −20 °C upon arrival at the laboratory (University of Guelph, Guelph, ON). One biological replicate was selected per zone (*n* = 10) for further extraction. The eDNA sampler, filter housings, and forceps were decontaminated using a 5% bleach solution and rinsed thoroughly with deionized water before use. Sterile nitrile gloves were worn in the field when handling filter housings and gloves were changed for each sample. A field negative control was not included; however, the rigorous decontamination and sample collection protocols used with the OSMOS eDNA sampler minimize the risk of contamination at the sample collection stage and previous studies in our laboratory have demonstrated no detectable contamination of field blanks (*e.g*., Nolan et al., 2023). Immediately before DNA extraction, filters were placed on uncovered, sterile petri dishes using sterile forceps (a fresh pair for each filter) and bisected with a factory-sterilized razor blade (discarded after a single use); the filters remained uncovered for several minutes until the residual ethanol evaporated. An unused filter was included as a negative control of DNA extraction (‘extraction blank’); this filter was handled with forceps, placed on an uncovered petri dish, bisected with a razor blade, and remained uncovered for an equal length of time as the samples. Sterile nitrile gloves were worn during filter manipulation and a new pair was used for each filter to reduce the risk of cross-contamination; gloves were changed away from the laboratory bench.

### DNA extraction

DNA extraction was conducted on half of each filter using the NW kit according to the manufacturer’s protocol (https://www.mn-net.com/media/pdf/ee/11/43/Instruction-NucleoSpin-eDNA-Water.pdf). The remaining half of each filter was processed using the BT kit using the manufacturer’s protocol for the purification of total DNA from animal tissue and the following modifications to optimize the protocol for filter samples (Milián-García, 2022): each half-filter was bisected, and each quarter-filter was extracted independently to accommodate the size of the microcentrifuge tube. This step aims to avoid overloading the microcentrifuge tube during the lysis stage and the spin column during the isolation stage. Prior to extraction with the BT kit, quarter-filters were further processed into thin strips with a sterilized razor blade (Milián-García et al., 2023). This step aims to avoid folding the filter to improve filter contact with the lysis buffer in the microcentrifuge tube and increase the recovery of DNA; this may not be necessary if larger tubes are used, however, we used the tubes included in the BT kit. Additional handling of the filters increases the risk of contamination and loss of yield (due to probable transfer of eDNA to the razor blade); however, we took reasonable precautions to reduce the risk of contamination (*i.e*., sterile instruments and glove changes) and the amount of eDNA loss was expected to be minimal. Next, (manufacturer step 1) the filter strips were placed in 2 mL microcentrifuge tubes with 250 mg of glass beads and 380 μL buffer ATL before homogenization for 1 min at 30 Hz using the TissueLyser (Qiagen, Hilden, Germany); (step 3) 400 μL buffer AL was added and vortexed, samples were incubated for 10 min at 56 °C, 400 μL 100% ethanol was added and vortexed; lysate was then transferred to the DNeasy Mini spin columns, centrifuged, flowthrough discarded, and the two washing steps conducted without further modifications. After the second wash (step 6), samples were centrifuged for 5 min at 20,000× *g*; (step 7), 100 μL buffer AE (prewarmed at 70 °C) was added, samples were incubated at room temperature on the bench-top for 15 min, and centrifuged for 5 min at 11,000× *g* to elute. Following DNA extraction with the BT kit, samples from each quarter-filter (*n* = 20) were pooled to produce a single DNA sample per half-filter (*n* = 10). As stated in the published protocol, half-filters may be used instead of quarter-filters to halve the number of extractions and the associated cost; however, in some cases a reduction in total yield may be observed when using half-filters. For example, samples containing greater quantities of organic matter and/or DNA may saturate the BT kit spin column when a greater portion of the filter is used for a single extraction. Therefore, quarter-filters were used here to reduce the possibility of saturating the columns to maximize the sensitivity of target species detection in low-concentration samples using downstream analytical methods. DNA extractions were performed in a clean eDNA laboratory space using filtered pipette tips and wearing sterile nitrile gloves.

### DNA visualization and quantification

The success of DNA extraction was evaluated in two ways. DNA was separated on a 1% agarose gel prepared with 1X TBE buffer and compared to the GeneRuler 1kb+ DNA ladder (Thermo Fisher Scientific, Waltham, MA, USA). Total dsDNA concentration (ng/μL) was quantified using the Qubit™ dsDNA Broad Range Assay kit and Qubit™ 4 Fluorometer (Thermo Fisher Scientific, Waltham, MA, USA) according to the manufacturer’s protocol. Three measurements were taken 10 min apart for each DNA extract. Measurements were repeated using the Qubit™ dsDNA High Sensitivity Assay kit for samples below the limit of quantification of the Broad Range assay.

### Quantitative PCR (qPCR) and DNA purification

qPCR was conducted using the TaqMan brook trout assay *BRK2* (Wilcox et al., 2013), which targets a 140 bp region of *cytochrome oxidase b* (*cyt b*) encoded by the mitochondrial genome. The *BRK2* assay uses a species-specific primer set (F: 5′-CCACAGTGCTTCACCTTCTATTTCTA-3′; R: 5′-GCCAAGTAATATAGCTACAAAACCTAATAGATC-3′) and probe (5′-ACTCCGACGCTGACAA-3′) with FAM reporter and non-fluorescent minor groove-binding quencher (NFQ-MGB).

Amplification was performed at the Genomics Facility’s Advanced Analysis Centre (AAC) at the University of Guelph (Ontario, Toronto, Canada) on the StepOnePlus™ Real-Time PCR System (Thermo Fisher Scientific, Waltham, MA, USA). Only samples extracted using the BT kit (*n* = 10) were initially analyzed to test the qPCR protocol while preserving the lower-volume NW extracts. A 10 µL reaction was prepared for each BT kit extracted sample in triplicate, containing 3.75 µL of template DNA, 5 µL of Master Mix from the SensiFAST™ Probe Hi-ROX kit (Meridian Biosciences, Cincinnati, Ohio, USA), 1 µL of mixed forward and reverse primer (5 µM stock), and 0.25 µL of probe (10 µM stock). Samples were run alongside six standards of synthetic gBlock™ (Integrated DNA Technologies, Coralville, Iowa, USA) of the amplicon prepared by serial 10-fold dilutions (concentration ranging from 3,860,000 to 38.6 copies/µL), an extraction blank (described above), and a no-template control (NTC), each in triplicate. A standard curve was created using the six serially diluted standards. The following reaction conditions were used: 4 min at 95 °C, and 40 cycles of 10 s at 95 °C and 30 s at 60 °C. Samples with one or more positive replicates were classified as positive detections. Cycle threshold (C_t_) values were determined using StepOne™ Software version 2.3 (Applied Biosystems, Waltham, MA, USA) and used to estimate target DNA concentrations (copies/µL) with reference to the standard curve. The limit of detection (LOD) and limit of quantification (LOQ) were calculated using the discrete threshold method (Klymus et al., 2020) with three replicates for each standard concentration. Importantly, as outlined by Klymus et al. (2020), positive detections below the LOD were considered meaningful, true positive detections but treated as qualitative data, as were all detections below the LOQ. When detections occur below the LOD, this simply indicates that there is less than 95% confidence in these low-concentration detections.

Magnetic bead DNA purification was performed on all samples (BT and NW kit extracts) using the NucleoMag PCR kit (Macherey-Nagel, Düren, Germany) according to the manufacturer’s protocol, with a starting volume of 10 µL and a final elution volume of 27.5 µL. Following purification, qPCR was repeated on all purified samples as described above, again in triplicate and including the extraction blank. Importantly, purified extracts were only analyzed using qPCR. In contrast, unpurified extracts were analyzed using ddPCR and metabarcoding since the evidence of inhibition in the preliminary qPCR run was not assumed to have an equal effect across analysis methods (*i.e*., ddPCR and metabarcoding).

### Droplet digital PCR (ddPCR)

ddPCR was conducted at the Advanced Analysis Centre (AAC) of the Genomics Facility at the University of Guelph (Ontario, Toronto, Canada) using the same brook trout assay described above for qPCR. Reactions were prepared to a total volume of 22 μL, containing 5 μL of template DNA, 5.5 μL 4X Q200 ddPCR Multiplex Supermix (Bio-Rad, Hercules, CA, USA), 0.5 μM of each primer, 0.25 μM of probe, and 9.85 μL of DNase/RNase-free ddH_2_O. Nanolitre-sized droplets were generated on the Automated Droplet Generator Instrument (Bio-Rad, Hercules, CA, USA). Amplification was conducted on the C1000 Touch Thermal Cycler (Bio-Rad, Hercules, CA, USA) under the following reaction conditions: 10 min at 95 °C; 40 cycles of 30 s at 94 °C, 30 s at 60 °C, and 1.5 min at 72 °C; followed by 10 min at 98 °C. Three technical replicates were run per sample, including the extraction blank (described above). Samples were run alongside synthetic gBlock™ (Integrated DNA Technologies, Coralville, Iowa, USA) of the amplicon and a no-template control (NTC). A manual threshold was set to exclude positive signal detected in the extraction blanks (Kokkoris et al., 2021).

### DNA metabarcoding

DNA metabarcoding was conducted at the Agriculture & Food Laboratory at the University of Guelph (Ontario, Toronto, Canada) following the methods described in the original publication of the MiFish primer sets (Miya et al., 2015) and the 16S Metagenomic Sequencing Library Preparation Guide (Illumina, 2013). Briefly, amplicon sequencing libraries were prepared by PCR run in triplicate using the MiFish U&E primer sets for fishes, which target a 163–185 bp region (∼220 bp amplicon including primer regions) of the 12S rRNA gene. Library quality and quantity were assessed by a Fragment Analyzer Automated CE System with the dsDNA 935 reagent kit (Agilent Technologies, Santa Clara, CA, USA) and Qubit™ Fluorometer with the Qubit™ dsDNA Broad Range Assay kit (Thermo Fisher Scientific, Waltham, MA, USA). Purified libraries were normalized and combined in an equal molar ratio. Sequencing was conducted using a MiSeq sequencer with a MiSeq Reagent Kit v2 (Illumina) with 2 × 250 paired-end cycles according to the manufacturer’s protocol. PhiX (Illumina) was included as an internal sequencing control, and a fish control containing ten species was included as a positive control for the entire library preparation and sequencing process.

Raw sequence reads were filtered using the MiSeq Sequencer System Software (Illumina) to remove low-quality sequences and trimmed to remove adaptor sequences. The remaining bioinformatics analysis was conducted using the MiFish Pipeline (Sato et al., 2018), which analyzes raw sequence reads to generate a species list. Parallel analyses using two additional bioinformatics pipelines–referred to as MetaWorks and Geneious pipelines–were conducted to validate the results of the MiFish Pipeline and assess congruence across pipelines. Over thirty-two available bioinformatic pipeline options for analyzing metabarcoding data are currently recognized (Hakimzadeh et al., 2023). The selection of a bioinformatic pipeline is closely tied to the user’s needs and level of informatics expertise. Across these available pipelines, there are distinct differences in user-friendliness, cost, marker compatibility (*i.e*., the gene(s) and gene region(s) which can be analyzed), scalability (*e.g*., the number of samples which can be analyzed simultaneously), and taxonomic assignment methods (including the reference database and classification approach), among other factors. In the present study, three pipelines (*i.e*., MiFish Pipeline, MetaWorks, and Geneious pipeline) were selected to represent a range of each of the aforementioned factors while using the most comprehensive 12S reference databases available. Importantly, all three pipelines are able to analyze metabarcoding data for the target gene region (12S). The MiFish Pipeline and Geneious pipeline are user-friendly, not scalable, and assign sequences to taxa using a sequence similarity approach; however, each uses a different reference sequence database (see details below). MetaWorks requires bioinformatics expertise, is highly scalable, and assigns sequences to taxa using a sequence composition approach against a different reference sequence database to the other two pipelines (see details below). While the MiFish Pipeline and MetaWorks are available to use at no cost, the Geneious pipeline requires a paid Geneious Prime subscription license.

The use of multiple pipelines with different methodologies reduces the risk of false negatives and false positives resulting from the limitations associated with each method. The varied methodologies implemented across these three pipelines, especially concerning taxonomic assignment (see details below), allows the robustness of results to be evaluated. The performance of each pipeline was compared to assess the degree of congruence and identify any inconsistencies in detection success between pipelines arising from their respective limitations in the context of our dataset. Since taxonomic assignment is an especially variable and influential step in the analysis of metabarcoding reads (Mathon et al., 2021), positive detections of the target genus (*i.e*., *Salvelinus*) made using the integrated taxonomic assignment methods for the MiFish Pipeline and MetaWorks were further validated across additional taxonomic assignment methods (see details below).

#### MiFish pipeline

The MiFish Pipeline was conducted on the MitoFish online server (v1.00 2019; http://mitofish.aori.u-tokyo.ac.jp/mifish/) (Sato et al., 2018). This pipeline must be run independently and sequentially for each sample. The pipeline workflow and parameters are described by Sato et al. (2018). Default values were retained for all customizable parameters as follows: (1) filtering reads by length: read size was set to 229 +/− 25; (2) usearch: minimum read size for filtering was set to 10, and identity threshold for clustering was set to 0.99; (3) BLASTN: identity was set to 0.97. Taxonomic assignment was conducted using the MitoFish database (version 3.0, 7,565 fishes) (Sato et al., 2018) as an integrated part of the pipeline. Hits matching the target genus (*Salvelinus*) were validated by running a BLAST search (megablast) of the sequence with the highest number of reads against the NCBI GenBank database nucleotide collection (release 243, April 15, 2021).

#### MetaWorks pipeline

MetaWorks operates at the command line in a conda environment as one of several Snakemake pipelines that support a number of marker gene amplicons and metabarcodes (Porter & Hajibabaei, 2022). This pipeline can analyze raw sequence reads from an entire dataset in a single run rather than operating on a per-sample, iterative basis. MetaWorks (version 1.13.0). The parameters were as follows: (1) pairing (Phred score quality cutoff: 20; minimum overlap; 25, the maximum fraction of mismatches allowed in overlap: 0.02; minimum fraction of matching in overlap: 0.90); (2) trimming and filtering (minimum sequence length: 100 bp; error rate: 0.1; Phred quality score cutoffs at the ends: 20, 20; minimum adapter overlap: 3; maximum number of N’s: 3; (3) minimum number of reads per cluster: minsize 3; (4) marker classifier was set to ‘12S-fish’; (5) pseudogene filtering was set to ‘no’; 3) taxon refinement was set to ‘-e Vertebrata.’ Taxonomic assignment was conducted using the 12S fish-trained RDP classifier (Porter & Hajibabaei, 2022; Wang et al., 2007). MetaWorks generates an output file containing sequences for each zero-radius operational taxonomic unit (ZOTU) in multi-fasta format across all samples, which permits cross-database validation. Hits matching the target genus (*Salvelinus*) were validated by running a BLAST search (megablast) of the corresponding ZOTUs against the NCBI GenBank database nucleotide collection (release 243, April 15, 2021). Further validation was conducted by taxonomic assignment on the MIDORI server (http://www.reference-midori.info/server.php) using the integrated RDP classifier (Wang et al., 2007) against the MIDORI-curated ‘unique’ srRNA database with sequence coverage across the domain Eukarya.

#### Geneious pipeline

Geneious Prime 2021 (version 2021.1; Biomatters, Ltd., Auckland, New Zealand) was used to analyze raw sequence reads on a per-sample, iterative basis. The pipeline steps and key parameters for COI are described by Milián-García et al. (2021). Primers were trimmed using the Trim Ends > Trim Primers option immediately before merging paired reads. Taxonomic assignment was conducted by exporting the consensus sequence from each OTU into a single multi-fasta file per sample and performing a BLAST search (megablast) against the NCBI GenBank database nucleotide collection (release 243, April 15, 2021). For each sample, the hit tables for all OTUs were downloaded as XML files and imported into Geneious to compile and filter the hits for all OTUs of a particular sample.

### Statistical analysis

A correction factor was applied across the raw results from all analysis methods (*i.e*., Qubit™ fluorometry, qPCR, ddPCR, and metabarcoding) to facilitate direct quantitative comparison of BT extracts to NW extracts. This correction factor accounts for the difference in total elution volumes per half filter between kits: 200 µL for BT extracts and 100 µL for NW extracts (NW extracts are 2X concentrated compared to BT extracts). The results presented hereafter were corrected by multiplying the raw DNA concentration (for Qubit™ fluorometry, qPCR, and ddPCR) and raw number of reads assigned to brook trout (for metabarcoding) for BT extracts by 2. The data were cleaned by assigning ‘NA’ when the DNA concentration could not be determined for an extract (*i.e*., qPCR: Ct value “undetermined”) to exclude these extracts from further analysis; however, zero values were retained for ddPCR. Shapiro-Wilk normality tests (‘stats’ package version 4.1.1; R Core Team, 2024) were conducted in RStudio™ (2,023.03.0 + 386) using R (4.1.1; R Core Team, 2024) at a significance level (α) of 0.05 to assess the normality of the following datasets: total dsDNA yield determined by Qubit™ fluorometry (mean across triplicate readings), target DNA concentration determined by qPCR and ddPCR (mean across technical triplicates), respectively, and the number of reads assigned to brook trout using the MiFish Pipeline and MetaWorks, respectively. Paired t-tests (‘stats’) or Wilcoxon signed-rank tests (non-parametric) (‘stats’) were then conducted as appropriate in RStudio™ at a significance level (α) of 0.05 to compare the BT and NW kits based on the abovementioned performance metrics. Box plots and bar plots (‘ggplot2’ package version 3.5.0; (Wickham, 2016)) were created in RStudio™ to visualize the comparisons of target DNA concentration (for qPCR and ddPCR), and number of reads assigned to brook trout (for metabarcoding with two of the chosen bioinformatics pipelines).

A *post-hoc* power analysis (‘pwrss’ package version 0.3.1; R Core Team, 2024) and effect size calculation (Hedges’ g and 95% confidence interval (CI) using un-pooled standard deviation (SD); ‘effectsize’ package version 1.0.1; Ben-Shachar, Lüdecke & Makowski, 2020) were conducted in RStudio™ (2,023.03.0 + 386) using R to assess the power (β) and effect size (g) of each paired t-test and Wilcoxon signed-rank test performed. The results of these *post-hoc* analyses are intended to inform the interpretation of the results of the current study. Further, an *a priori* power analysis was conducted in RStudio™ (2,023.03.0 + 386) using R to determine the minimum sample size required to achieve 80% power at the established effect size for each paired t-test and Wilcoxon signed-rank test performed. The minimum sample sizes determined by these *a priori* analyses are intended as a guide for similar studies.

## Results

An overall comparison of cost, time of extraction, and performance of the BT and NW kits is presented in Table 1.

**Table 1:** Comparison of cost, time, and performance specifications for the DNeasy® Blood & Tissue kit (Qiagen, Hilden, Germany) and NucleoSpin® eDNA Water kit (Macherey-Nagel, Düren, Germany). All specifications for the DNeasy® Blood & Tissue kit represent two separate extractions for each sample, as conducted in the current study. The cost per sample was calculated using the largest kit size available. Time specifications were established from the modified protocol (Milián-García, 2022) for the DNeasy® Blood & Tissue kit and the manufacturer’s protocol for the NucleoSpin® eDNA Water kit. Total dsDNA yield from filtered environmental water samples (*n* = 10) was determined using the Qubit™ dsDNA Broad Range and High Sensitivity Assays and the Qubit™ 4 Fluorometer (Thermo Fisher Scientific, Waltham, MA, USA).

Specification		DNeasy® Blood & Tissue kit	NucleoSpin® eDNA Water kit
Cost	Cost per sample (CAN)	$10.52	$15.82
	Additional materials required per sample	1.5, 2 mL microcentrifuge tubes (x2)	1.5, 5, 15 mL centrifuge tubes, NucleoSpin® Filter Midi
Time	Filter processing into strips	Yes	No
	Incubation time (min)	25 and overnight	16
	Centrifugation time (min)	14	12
	No. pipetting steps per sample	16	13
Performance	Total dsDNA yield (mean ± SD; ng/µL)	2.26 ± 1.38	1.56 ± 0.83
	Samples with positive detection of *S. fontinalis* (%) by qPCR*	70%	70%
	Samples with positive detection of *S. fontinalis* (%) by ddPCR	90%	70%
	Samples with positive detection of *S. fontinalis* (%) by metabarcoding	100%	100%

**Note:**

* Only the results of qPCR on the purified samples are reported.

### Cost

The cost per sample (CAD/USD) for the BT and NW kits are $10.52/$8.40 and $15.82/$12.63, respectively, at the time of the study (2021). These costs reflect the DNA extraction protocols used in the current study (*i.e*., two parallel extractions per sample using the BT kit and one extraction per sample using the NW kit). Notably, the cost per extraction for the BT kit ($5.26/$4.20) is one-third of the cost for the NW kit. The additional consumable supplies required for the BT kit are two 1.5 mL and two 2 mL microcentrifuge tubes per sample (doubled to account for two extractions per sample). The NW kit requires one 5 and 15 mL centrifuge tube and one NucleoSpin® Filter Midi per sample. Additional costs are incurred for these required supplies as they are not included in their respective kits; however, these additional costs were not considered in the current comparison as they are minimal and vary between distributors.

### Time

The total incubation time required for the NW kit extraction is 16 min, while the modified protocol for the BT kit includes overnight incubation and a second 25-min incubation. The total centrifugation times for the BT and NW extraction kits are 14 and 12 min, respectively. The BT kit extraction involves 16 reagent additions or transfers of the sample by pipetting, while the NW kit extraction involves 13 of these pipetting steps. Again, the number of reagent additions for the BT kit was doubled to account for the two extractions conducted per sample.

### Total dsDNA yield

The mean total dsDNA concentration (ng/µL) using the BT kit and the NW kit is reported in Table 1. There was no significant difference in total dsDNA concentration (ng/µL) between the two kits (means, BT kit: 2.3 ng/μL *vs*. NW kit: 1.6 ng/μL; paired t-test; t(9) = 2.18; *p* = 0.0567; power = 25%). The effect size, measured by Hedges’ g, was moderate (g = 0.58, 95% CI [−0.29 to 1.43]), indicating a meaningful difference between kits.

### qPCR

#### Standard curves, LOD and LOQ

The equation of the standard curve run alongside the unpurified BT kit extracts is given as y = −3.3763x + 38.119 (R^2^ = 0.9997). The LOD and LOQ were 38.6 copies/µL, which is the concentration of the lowest-concentration standard used. The estimated LOD and LOQ may be lower if an additional lower-concentration standard (*i.e*., 3.86 copies/µL) was used. The equation of the standard curve run alongside the purified BT and NW kit extracts is given as y = −3.4912x + 38.563 (R^2^ = 0.9997). The LOD and LOQ was 386 copies/µL. The estimated LOD and LOQ may be lower if a greater number of replicates of each standard were run.

#### Target species detection

**Figure 2:**
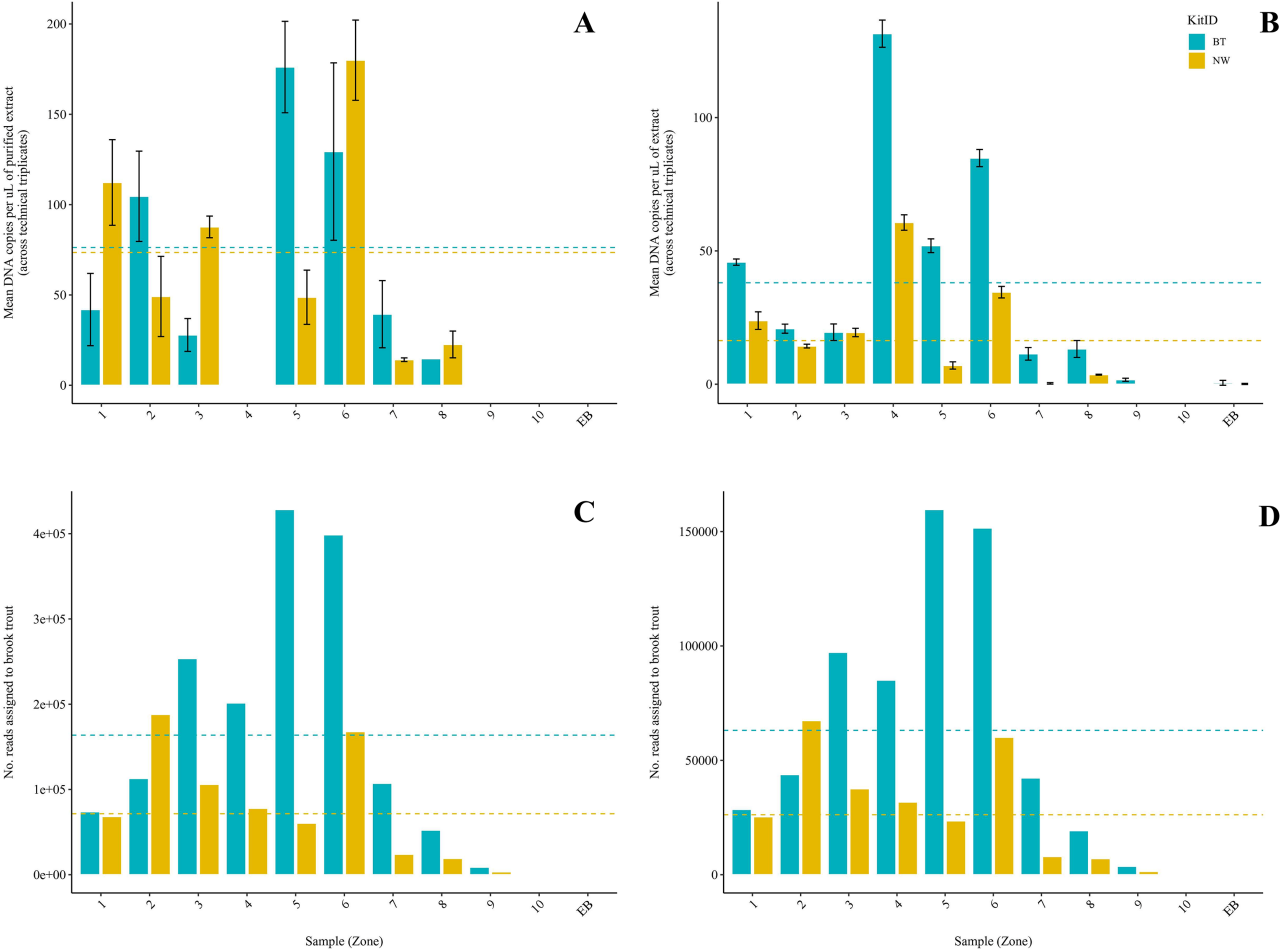
Comparison of two DNA extraction methods based on detection of brook trout (*Salvelinus fontinalis*) eDNA by qPCR, ddPCR, and metabarcoding. DNA extraction was conducted on each sample (*n* = 10) in parallel using the DNeasy Blood & Tissue kit (“BT”; Qiagen, Hilden, Germany) according to a modified in-house protocol designed for filters and the NucleoSpin eDNA Water kit (“NW”; Macherey-Nagel, Düren, Germany) according to the manufacturer’s protocol. Mean copies of DNA per µL were determined by qPCR (post magnetic bead purification) (A) and ddPCR (B) using the BRK2 assay. Values are shown as mean (calculated across technical triplicates) ± SD. Reads assigned to *S. fontinalis* were determined by the MiFish Pipeline (C) and MetaWorks (D) following metabarcoding using the MiFish-U&E primer sets. Dashed lines represent means (calculated across samples, *n* = 10) for each DNA extraction method.

**Figure 3:**
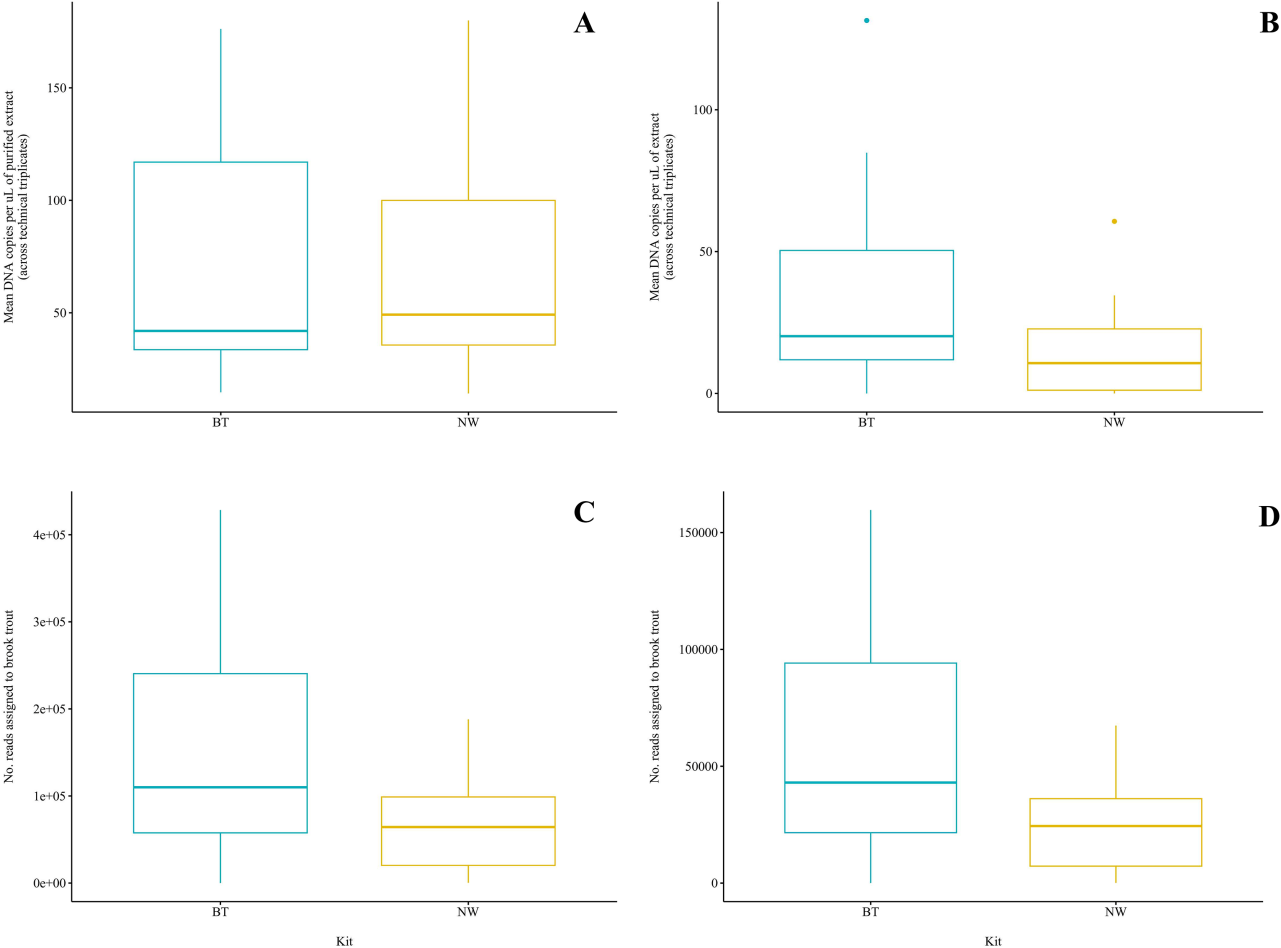
Comparison of two DNA extraction methods based on detection of brook trout (*Salvelinus fontinalis*) eDNA by qPCR, ddPCR, and metabarcoding. DNA extraction was conducted on each sample (*n* = 10) in parallel using the DNeasy Blood & Tissue kit (“BT”; Qiagen, Hilden, Germany) according to a modified in-house protocol designed for filters and the NucleoSpin eDNA Water kit (“NW”; Macherey-Nagel, Düren, Germany) according to the manufacturer’s protocol. Mean copies of DNA per µL were determined by qPCR (post magnetic bead purification) (A) and ddPCR (B) using the BRK2 assay. Reads assigned to *S. fontinalis* were determined by bioinformatic analysis with the MiFish Pipeline (C) and MetaWorks (D) following metabarcoding using the MiFish-U&E primer sets. The DNA extraction methods did not significantly differ based on detection by qPCR (t(6) = 0.1019; *p* = 0.92) or metabarcoding using the MiFish Pipeline for bioinformatic analysis (t(9) = 2.225; *p* = 0.0531); however, the BT kit was significantly favoured based on detection by ddPCR (V = 45; *p* = 0.00915) and metabarcoding using MetaWorks for bioinformatic analysis (t(9) = 2.381; *p* = 0.0411).

Brook trout DNA was amplified in a single BT kit extract (collected at Zone 9; two positive replicates of three; mean: 3.88 copies/µL) before magnetic bead DNA purification (NW kit extracts were not included in this run). The remaining nine unpurified BT kit extracts did not amplify. The standards amplified as expected and there was no amplification in the extraction blank or the NTC. Following purification, brook trout DNA was amplified in seven (70%) BT kit extracts and seven (70%) NW kit extracts representing the same samples (Fig. 2A). This clear improvement in detection success following purification of the BT kit extracts strongly suggests the presence of PCR inhibitors in the samples before purification. Target DNA concentrations (copies/µL) for each technical replicate and mean target DNA concentrations for each extract are reported in Table 2. The standards amplified as expected and there was no amplification in the extraction blank from either of the kits or the NTC. In general, samples amplified at relatively high cycle numbers (30 ≤ Ct ≤ 37), and the concentration of brook trout DNA in these samples challenged the LOD and LOQ of the assay. There was no significant difference in target DNA concentration (mean across technical triplicates) determined by qPCR between the two kits (BT kit: 76.3 copies/μL *vs*. NW kit: 73.5 copies/μL) (paired t-test; t(6) = 0.1019; *p* = 0.92; power = 5.1%) (Fig. 3A). The effect size, measured by Hedges’ g, was extremely small (g = 0.04, 95% CI [−0.94 to 1.02]), indicating no practical difference between kits.

**Table 2:** Comparison of two DNA extraction methods based on detection of brook trout (*Salvelinus fontinalis*) eDNA by qPCR (post magnetic bead purification) and ddPCR. DNA extraction was conducted using the DNeasy Blood & Tissue kit (“BT”; Qiagen, Hilden, Germany) according to a modified in-house protocol designed for filters and the NucleoSpin eDNA Water kit (“NW”; Macherey-Nagel, Düren, Germany) according to the manufacturer’s protocol.

Site	Zone	Kit	qPCR (copies/µL)					ddPCR (copies/µL)				
			Technical replicate			Mean	SD	Technical replicate			Mean	SD
			1	2	3			1	2	3		
Creek 1	1	BT	20.5	45.1	60.2	41.9	20.0	46.1	46.8	44.5	45.8	1.2
		NW	127.6	124.3	85.0	112.3	23.7	27.3	23.6	20.7	23.9	3.3
	2	BT	99.4	82.7	131.8	104.6	25.0	20.2	19.5	22.8	20.8	1.7
		NW	52.3	69.6	25.5	49.2	22.2	14.1	15.1	13.7	14.3	0.7
	3	BT	U	21.4	34.3	27.8	9.1	20.6	16.0	21.9	19.5	3.1
		NW	94.6	84.1	84.3	87.7	6.0	21.2	18.7	18.4	19.4	1.6
	4	BT	U	U	U	–	–	130.2	137.1	127.2	131.5	5.1
		NW	U	U	U	–	–	61.8	62.8	57.4	60.7	2.9
	5	BT	167.0	204.7	156.8	176.2	25.3	49.0	53.6	53.3	51.9	2.6
		NW	38.2	65.9	42.2	48.7	15.0	8.4	5.6	7.1	7.0	1.4
	6	BT	186.0	104.3	97.9	129.4	49.1	82.3	83.7	88.4	84.8	3.2
		NW	204.4	174.4	161.1	180.0	22.2	32.2	36.5	34.9	34.5	2.2
Creek 2	7	BT	43.5	55.6	19.0	39.4	18.6	8.9	13.6	11.7	11.4	2.4
		NW	13.5	14.8	U	14.2	1.0	0.00	0.45	0.51	0.32^‡^	0.3
	8	BT	14.7	U	U	14.7	–	10.3	16.6	12.8	13.2	3.2
		NW	24.5	28.8	14.5	22.6	7.4	3.7	3.5	-^†^	3.6	0.2
	9	BT	U	U	U	–	–	2.2	1.1	1.7	1.7	0.6
		NW	U	U	U	–	–	0.00	0.00	0.00	0.00	–
	10	BT	U	U	U	–	–	0.00	0.00	0.00	0.00	–
		NW	U	U	U	–	–	0.00	0.00	0.00	0.00	–
	EB	BT	U	U	U	–	–	0.00	1.6	0.00	0.53	0.9
		NW	U	U	U	–	–	0.00	0.00	0.36	0.12	0.2

**Notes:**

U: brook trout undetected.

EB: extraction blank.

† Technical duplicate was run for this extract only, instead of triplicate (necessary in order to include the NTC).

‡ Below the set threshold (0.53 copies/μL).

### ddPCR

Brook trout DNA was amplified in nine (90%) BT extracts and seven (70%) NW extracts above the set threshold (described below) (Fig. 2B). Target DNA concentrations (copies/µL) for each technical replicate and mean target DNA concentrations for each extract are reported in Table 2. The positive control amplified as expected and there was no amplification (zero positive droplets) in the NTC. However, amplification was observed in the extraction blank for the BT and NW kit extracts in one replicate each (two positive droplets and one positive droplet, respectively); this amplification indicates low-level contamination. A threshold was set at 0.53 copies/μL across all sample means to exclude positive signal from the extraction blanks (the maximum level of detection in either extraction blank was 0.53 copies/μL, calculated as the mean across technical triplicates), which identified one NW kit extract (collected at Zone 7) as a false positive as target DNA was detected below the threshold level. Target DNA concentration (mean across technical triplicates) determined by ddPCR was significantly greater for BT kit extracts (40.8 copies/μL) than NW kit extracts (18.1 copies/μL) (Wilcoxon signed-rank test; V = 45; *p* = 0.00915; power = 37%) (Fig. 3B). The effect size, measured by Hedges’ g, was moderate (g = 0.62, 95% CI [−0.25 to 1.47]), indicating a meaningful difference between kits.

### DNA metabarcoding

Amplification was observed in all samples during library preparation. Spurious amplification was observed in the extraction blanks. Sequencing on the Illumina MiSeq system produced a total of 3,309,729 paired-end reads with a mean length of 216 bases across 20 samples. The mean number of raw reads per sample extracted with the BT kit was 188,309 (min. 120,484, max. 367,508) *vs*. 142,664 (min. 74,741, max. 257,974) per sample extracted with the NW kit, excluding the extraction blanks. The mean number of raw reads across the two extraction blanks was 70,246, with a mean length of 100 bases.

Brook trout eDNA was successfully detected in each of the 20 samples using each of the three pipelines: (1) the MiFish Pipeline (Fig. 2C), (2) MetaWorks (Fig. 2D), and (3) the pipeline implemented in Geneious Prime. The number of target reads determined using the MiFish Pipeline and MetaWorks for each extract are reported in Table 3. When the taxonomic assignment was conducted using a BLAST search against the NCBI GenBank or MitoFish databases, brook trout matched with 100% identity. When the taxonomic assignment was conducted using the RDP classifier trained against the MIDORI database, brook trout matched with a perfect confidence score (=1). Similarly, brook trout DNA was detected using the 12S_fish RDP classifier as part of the MetaWorks pipeline with 100% bootstrap support. Consequently, the genus *Salvelinus* also matched with a perfect confidence score (=1). Brook trout eDNA was detected with 100% identity and a perfect confidence score (=1) in both extraction blanks using the three pipelines and corresponding taxonomic assignment methods. There was no significant difference between the BT and NW kits based on the number of reads assigned to brook trout using the MiFish pipeline (means, BT kit: 163,696.8 reads *vs*. NW kit: 71,510.2 reads) (paired t-test; t(9) = 2.225; *p* = 0.0531; power = 38%) (Fig. 3C). The effect size, measured by Hedges’ g, was large (g = 0.73, 95% CI [−0.15 to 1.59]), indicating a substantial difference between kits. However, the number of reads assigned to brook trout was significantly greater for BT kit extracts (mean, 63,092.6 reads) than NW kit extracts using MetaWorks (mean, 26,212.9 reads) (paired t-test; t(9) = 2.381; *p* = 0.0411; power = 42%) (Fig. 3D). The effect size, measured by Hedges’ g, was large (g = 0.78, 95% CI [−0.11 to 1.64]), indicating a substantial difference between kits.

**Table 3:** Comparison of two DNA extraction methods based on detection of brook trout (*Salvelinus fontinalis*) eDNA by metabarcoding using the MiFish Pipeline and MetaWorks for sequence analysis. DNA extraction was conducted using the DNeasy Blood & Tissue kit (“BT”; Qiagen, Hilden, Germany) according to a modified in-house protocol designed for filters and the NucleoSpin eDNA Water kit (“NW”; Macherey-Nagel, Düren, Germany) according to the manufacturer’s protocol.

			No. target reads	
Site	Zone	Kit	MiFish pipeline	MetaWorks
Creek 1	1	BT	73,830	28,534
		NW	68,253	25,309
	2	BT	112,852	43,760
		NW	188,035	67,382
	3	BT	253,512	97,180
		NW	105,920	37,583
	4	BT	201,418	85,038
		NW	77,890	31,751
	5	BT	428,344	159,630
		NW	60,422	23,522
	6	BT	398,632	151,536
		NW	167,822	60,058
Creek 2	7	BT	107,208	42,274
		NW	23,967	7,958
	8	BT	52270	19,246
		NW	19,095	7,030
	9	BT	8,800	3,680
		NW	3,394	1,417
	10	BT	102	48
		NW	304	119
	EB	BT	156	44
		NW	215	78

**Note:**

EB: extraction blank.

### Minimum sample size estimation

Given the reported effect sizes, the minimum sample sizes required to achieve 80% power (β) at a 5% significance level (α) for each paired t-test or Wilcoxon signed-rank test conducted in the current study are as follows: total dsDNA yield: *n* = 44; qPCR: *n* = 7,270; ddPCR: *n* = 26; MiFish: *n* = 27; MetaWorks: *n* = 24. These sample sizes indicate the requirement for each group (*e.g*., the number of paired samples).

## Discussion

We evaluated the cost, time, and performance of a novel eDNA extraction kit against a high-performing kit commonly used for the extraction of eDNA captured on filters. We compared their performance based on total dsDNA yield, qPCR, ddPCR, and metabarcoding using three different bioinformatic analysis methods. We demonstrated that method optimization in eDNA studies, especially for the DNA extraction method, is critical to improving the quality of results while balancing the objectives and limitations of a particular study. Overall, we provide a case study using a small sample set to guide eDNA practitioners to conduct similar optimization studies and we present a preliminary dataset which we use to calculate effect sizes and minimum sample sizes (*i.e*., to achieve 80% power) for comparing two methods. The framework we established could be applied as novel kits become available, allowing practitioners to make informed methodological decisions over time by quickly comparing new methods against established methods in the field.

### Power analysis

The power of each paired t-test and Wilcoxon signed-rank test used to compare the two extraction kits was below the conventional 80% target (power ranged from 25% to 42%, with an outlier of 5.1% attributed to qPCR). Consequently, the type II error rate is greater than desired for each statistical test, which means that there is an increased probability of not detecting a significant difference that exists. The effect sizes ranged from extremely small to large across analytical methods and, similarly, the minimum sample sizes per group ranged from 24 to 44, and an outlier of 7,270 attributed to qPCR. We interpret our results accordingly, below, and discuss the implications of these findings in the current study for each analytical method in turn. Notably, the cost and time assessments included in our comparison of the kits is not impacted by the sample size. These results may be used to inform future study design in subsequent methods optimization efforts.

### Determination of the preferred DNA extraction kit

In the current study, the preferred DNA extraction kit was determined through the summation of cost, time, and performance (*i.e*., total dsDNA yield, brook trout detection success determined by qPCR, ddPCR, and metabarcoding). Detection success considered both the number of positive samples (*i.e*., samples in which brook trout was detected) and the amount of brook trout DNA detected (represented as the target DNA concentration for qPCR and ddPCR, and as the number of target reads for metabarcoding). A balanced assessment was taken to select the kit with the preferred combination of specifications. This assessment considered the following: (1) the ideal kit would have superior performance (*i.e*., detection success) across all three detection methods at a lower cost per sample and time of extraction; however, (2) where neither kit is the ideal kit, performance and cost specifications are prioritized over time of extraction; and (3) in the case that one kit has slightly lower performance than the other, this kit may be preferred if the cost is significantly lower. A similar or modified framework may be applied to future method optimization studies in accordance with study-specific goals and limitations and is not limited in its application to the method of DNA extraction. For example, a well-funded study comparing two qPCR assays for the detection of an invasive species may adjust the framework to favour the slightly more sensitive assay, despite a considerably higher cost.

### Comparison of DNA extraction kits

#### Performance

Both kits successfully extracted DNA from the filter samples, evidenced by Qubit™ fluorometry. The total dsDNA concentration did not significantly differ between kits. The effect size was moderate, which indicates that a significant difference may have been found with a larger sample size (*e.g*., 44 extracts per kit, the minimum recommended sample size for 80% power). Overall, the amount of brook trout DNA was assumed not to differ significantly between BT kit and NW kit extracts of the same sample unless patchy distribution of the target species eDNA on the filter occurred. The latter can affect the ability to identify species from filter fragments instead of whole filters, especially when their DNA is expected to appear in low concentrations (Milián-García et al., 2023). In the present study, differential detection success was only suspected due to differences in inhibitor removal across kits, as the NW kit has an integrated inhibitor removal protocol which the BT kit lacks. Samples extracted using both kits were purified post-extraction using magnetic beads to reduce the effect of inhibition for qPCR but not for metabarcoding or ddPCR.

Brook trout detection success was equal across kits for metabarcoding (100%) and qPCR (70%); however, brook trout DNA was detected by ddPCR in nine (90%) BT kit extracts, while only in seven (70%) NW kit extracts (overall success rate of 80%). We detected significant differences between the kits using ddPCR (moderate effect size) and metabarcoding using MetaWorks (large effect size), which indicates a meaningful and substantial difference between the kits for these analytical methods with practical implications; in both cases, the BT kit outperforms the NW kit. In contrast, we did not detect significant differences between the kits using qPCR (extremely small effect size) and metabarcoding using the MiFish Pipeline (large effect size). These findings suggest that the kits do not differ in a practically meaningful way when qPCR is used for analysis in our study context; however, the opposite is indicated for metabarcoding using the MiFish Pipeline–subsequent studies may demonstrate a significant difference between these kits using this analytical method when a larger sample size is evaluated.

Overall, the BT kit was found to perform equally or better than the NW kit across analysis methods. This is somewhat unexpected, as the NW kit protocol involves an integrated inhibitor removal step which may be expected to improve detection success over the BT kit for samples with inhibitors present. However, the relative efficiency of this integrated inhibitor removal step compared to the magnetic bead DNA purification protocol is unknown. It is important to highlight that successful detection of the target species by qPCR in BT kit extracts was only observed after purification using magnetic beads (detection was observed in just one sample before purification).

In summary, the BT kit was considered the higher-performing kit according to our evaluation criteria. The BT kit led to detections in two additional samples by ddPCR over the NW kit and, on average, resulted in equal or significantly greater amounts of target DNA detected by qPCR, ddPCR, and metabarcoding. However, both kits were demonstrated to be effective in extracting eDNA with no statistically significant difference in total dsDNA concentration, though subsequent studies may find a significant difference with a larger sample size due to the moderate effect size identified in the current study.

#### Cost

The BT kit is clearly preferred over the NW kit in terms of cost. Even when the cost is doubled to account for two parallel extractions per sample according to the modified BT kit protocol, the cost of the NW kit is still nearly a third greater per sample. The cost of the BT kit could be halved if the efficacy of extracting DNA from a quarter filter is comparable to that of a half filter in this context. Our laboratory has collected evidence demonstrating successful extraction from half-filters in similar eDNA metabarcoding-based biomonitoring/biosurveillance studies (Milián-García et al., 2023; Pérez-Fleitas et al., 2023) with certain sample sets; validation of the half-filter method using the sample set of interest would be required to ensure that total DNA yields are not reduced, since the use of half-filters increases the risk of saturating the spin column. Despite this potential for cost reduction, it is essential to note that the BT kit might require a subsequent purification using magnetic beads to remove inhibitors before successfully detecting the target species using qPCR. This extra step represents additional cost and time. Furthermore, the BT kit protocol includes bead beating using the Qiagen TissueLyser. This presents an added cost to laboratories that do not already have this equipment, which was not accounted for in the cost estimation presented here. Therefore, the availability of equipment required for extraction using either the BT or NW kits should be considered when selecting the preferred kit for a certain laboratory.

#### Time

The NW kit is slightly less time-intensive than the BT kit. The BT kit requires a slightly higher centrifugation time and three additional pipetting steps; however, the BT kit requires a minimum of 1–3 h of lysis, up to overnight, and a second 10-min incubation in a second lysis buffer. Finally, the modified protocol includes a final 5-min incubation following the addition of the elution buffer before centrifugation. This total incubation time is much greater than the 16-min incubation time indicated in the NW kit protocol. Therefore, the NW kit is preferred in terms of extraction time. However, the greater incubation duration in the BT kit may contribute to its superior performance. Notably, the time spent cutting the filters into thin strips before extraction with the BT kit was not accounted for and is not insignificant, and strongly varies depending on the experimental experience of the researcher conducting the extraction and the number of filters to cut. This step was undertaken only for the BT kit to increase surface contact of the filter with the lysis buffer and proteinase K, as an addition to the manufacturer’s protocol, and not the NW kit, which has larger tubes designed to accommodate whole filters. However, additional filter manipulations increase contamination probability.

#### Cumulative assessment

When taken together, the performance, cost, and time specifications of the BT kit are preferred over the NW kit in our experimental context. The BT kit outperformed the NW kit in the current study at a fraction of the cost per sample. The high cost of the NW kit was not justified by its performance, and it was only preferred over the BT kit due to the shorter extraction time, including a considerably shorter incubation time. Importantly, the time savings that comes with the NW kit is hands-off time and is therefore not a strong motivator for using this kit over the BT kit. Future investigations may focus on further reducing the time and cost of the BT kit by identifying and further validating high-performing alternatives to performing two parallel extractions on each sample (*e.g*., a single extraction using one half-filter) across studies and laboratories.

### Comparison of analytical methods: qPCR, ddPCR, metabarcoding

The detection success rate of metabarcoding was 100% across all pipelines and databases used for taxonomic assignment, while ddPCR and qPCR had overall success rates of 80% and 70%, respectively. The superior sensitivity of ddPCR over qPCR has been demonstrated in the literature (Doi et al., 2015; Nathan et al., 2014). ddPCR has also been found to be less susceptible to inhibition and interference by non-target DNA than conventional PCR (applicable to PCR-based metabarcoding as conducted in this study) or qPCR (Basu, 2017; Hindson et al., 2013; Zhao et al., 2016). Interestingly, metabarcoding detected the target species in 100% of samples, which suggests that metabarcoding is more sensitive than ddPCR despite the presence of inhibitors. The reduced susceptibility of ddPCR to inhibition may explain the overall greater detection success by ddPCR than by qPCR, especially without inhibitor removal, but even when the inhibition effect is diminished by DNA purification before qPCR in the current study. However, the DNA purification process conducted before qPCR may have resulted in the loss of some DNA along with unwanted inhibitors, which may reduce the quantity of target DNA. The recovered DNA was also concentrated during the purification process, as the input volume was greater than the elution volume. In the case of metabarcoding, the two-step PCR approach used during library preparation may bias the amount of target species DNA present during sequencing which complicates comparisons of detection success to qPCR and ddPCR. Finally, the primers and target gene used for qPCR and ddPCR differ from those used for metabarcoding which may influence the efficiency of target species DNA amplification and consequently, the success of detection. Overall, caution should be applied when comparing rates of detection success across analytical methods in the present study due to differences in pre-processing of the extracts and inherent biases introduced by each analytical workflow.

Interestingly, the target DNA concentrations determined using qPCR and ddPCR were inconsistent for some extracts, meaning that relatively high or low amplification of an extract with qPCR or ddPCR was not necessarily corroborated by the other method. These instances of non-corroboration are not explained by differences in susceptibility to inhibition and/or interference of non-target DNA, as the expected target DNA concentrations determined by ddPCR would be greater than those given by qPCR, which is not always the case. It is possible that purification of the extracts before qPCR, but not ddPCR, may explain these inconsistencies. Altogether, this suggests the need for further optimization of the quantitative approaches, particularly qPCR, which was out of the scope of the present study.

### Comparison of metabarcoding analysis pipelines

Overall, brook trout was detected using all three metabarcoding analytical pipelines independently of the extraction kit used. The MiFish Pipeline resulted in the detection of brook trout with 100% identity in all 20 samples and both extraction blanks using the integrated taxonomic assignment method–a BLAST search of each OTU against the MitoFish database–and by exporting the OTU sequences and conducting a BLAST search against the NCBI GenBank database.

The MetaWorks pipeline also resulted in the detection of brook trout in all samples and extraction blanks using the integrated taxonomic assignment method–a trained 12S_fish RDP classifier–also matching the genus (*Salvelinus*) with a perfect confidence score (=1). Brook trout identity was also confirmed with 100% identity and a perfect confidence score (=1) when a representative sequence for each ZOTU was exported, a BLAST search was conducted against the NCBI GenBank database, and when an RDP classifier was used against the MIDORI database. Similarly, the pipeline implemented in Geneious Prime resulted in the detection of brook trout with 100% identity in all 20 samples and both extraction blanks by exporting the OTU consensus sequences and conducting a BLAST search against the NCBI GenBank database. Notably, the integrated BLAST feature in Geneious Prime failed to run to completion for most of the samples, potentially due to the search size, which poses a limitation for this particular pipeline. Conducting the same BLAST searches directly on the NCBI webpage rather than through Geneious Prime was successful.

The congruence of detection of the target species across the tested pipelines and taxonomic assignment methods supports the robustness of these bioinformatics methods for eDNA-based detection by metabarcoding. However, the scope of the current study is limited to a single target species and may not reflect the congruence of detection of other taxa and/or the whole community. Future comparisons of these and other existing pipelines on different datasets with various taxa of interest using different gene regions are warranted to identify optimal pipelines for bioinformatics analysis in the metabarcoding workflow. If such comparisons demonstrate congruence between pipelines, then pipelines which are more scalable, user-friendly, low or no cost, compatible with multiple markers, and less computationally demanding may be preferred and adopted as standard methods over less streamlined pipelines that produce the same results. Further, pipelines with output formats that better facilitate subsequent analysis and cross-validation may be preferred.

### Detection of target DNA in extraction blanks

Target DNA was detected at low levels in the extraction blank for each kit using ddPCR and metabarcoding (independent of the bioinformatic analysis method), but not using qPCR. The ultimate expectation is that the extraction blanks should be clean unless contaminated. When contamination is suspected, it is critical to determine the level of contamination, which stage of the workflow it was introduced, and whether it was sporadic or systemic (Sepulveda et al., 2020). The answers to these questions are essential to ensure the dataset is not compromised with massive cross-sample contamination that consequently undermines the reliability and utility of the dataset. Since contamination was not observed for qPCR, this suggests that contamination was introduced during laboratory workflows for ddPCR and metabarcoding at a stage following DNA extraction. Consequently, we consider these detections to be sporadic contamination rather than systemic, and we address each in the context of the respective analytical method (ddPCR or metabarcoding) accordingly.

To account for this contamination in our ddPCR results, we set a threshold equal to the greatest mean target DNA concentration detected in either extraction blank to exclude all positive detections in each extraction blank (Kokkoris et al., 2021). One extract (NW kit, Zone 7) was below this threshold and was identified as a false positive. Consequently, the corresponding rate of detection success (*i.e*., for the NW kit, analyzed using ddPCR) did not include this extract as a successful detection.

In the case of metabarcoding, we considered that, on average, the detection level in the extraction blanks was considerably lower than that of the samples (the mean number of reads assigned to brook trout in the extraction blanks was 185.5 *vs*. 117,603.5 in the samples). However, one particular extract (BT kit, Zone 10) had fewer reads assigned to brook trout than the extraction blank extracted with the same kit (102 *vs*. 156 reads), which may indicate false positive detection in this extract. Notably, low-level detection in negative controls is not infrequently observed in metabarcoding studies (25% of studies which included a negative control *vs*. ~6% for targeted detection) owing to the sensitivity of the method, and contamination is generally regarded as unavoidable (Sepulveda et al., 2020). Some studies apply thresholds to filter analytical samples based on the number of reads or their proportion according to the reads observed in the negative controls. However, these filtering thresholds are considered entirely arbitrary and this practice limits methodological standardization in the eDNA metabarcoding field. In addition to being arbitrary, any read count/proportion threshold is highly dependent on the methods used to generate and analyze a particular dataset. For example, the same amplicon sequence variants below the threshold that are discarded can pass this same threshold at higher sequencing depths or when relaxing quality filtration stringency during bioinformatics analysis. Also, any step along the sample processing analysis is a subsampling process, so establishing a threshold would add another unnecessary subsampling process that is highly variable among the different bioinformatic pipelines for data analysis. Based on the above considerations, no read subtractions from the analytical samples or threshold filtering based on the number of reads observed in the negative controls were applied here.

Overall, the low-level detection observed in the extraction blanks may reflect physical contamination and/or tag jumping–a computational source of error introduced during demultiplexing (a step following sequencing in the metabarcoding workflow), regardless of the analysis pipeline. Physical contamination may be more likely since tag jumping does not affect ddPCR analysis, and there were positive detections in the ddPCR-analyzed extraction blanks. However, it is possible that contamination was introduced through either source or a combination of sources. Importantly, the low level of detection observed in the extraction blanks relative to the analytical samples and the lack of consistent amplification in these blanks across all analytical methods suggest sporadic contamination of the blanks in their experimental context rather than systemic cross-contamination.

## Conclusions

No statistically significant difference between the BT and NW kits was found in brook trout detection by qPCR, nor by metabarcoding using the MiFish pipeline. However, the BT kit led to significantly greater brook trout detection by metabarcoding using MetaWorks and especially by ddPCR, where not only was the detected amount of target DNA greater for BT extracts, but an additional two samples were positive compared to the NW kit. According to our assessment approach, neither kit meets the criteria of an ideal kit, which is defined as having superior performance across all three detection methods at a lower cost per sample and time of extraction. Therefore, given these findings and under these particular experimental conditions, the BT kit was selected as the preferred method based on equal or superior performance at a lower cost, at the expense of prolonged procedures, assuming that the laboratories conducting the extractions have the necessary infrastructure. However, the NW kit may be favoured over the BT kit in laboratories lacking the infrastructure necessary for extractions with the BT kit, when faster extractions are prioritized despite a higher cost, or under different experimental conditions where the target species detection rates in any of the applied molecular analysis methods are significantly greater than those of the BT kit. All bioinformatic pipelines used to analyze the metabarcoding data detected brook trout in 100% of samples.

Our evaluation of methods is applicable to eDNA samples captured on filters and the findings should not be extrapolated to other sample types. This is a study-specific optimization; therefore, caution should be applied when generalizing the results herein to other taxa or sites. The analyses were conducted on small sample numbers, achieving less than 80% power, and without biological replication or a field blank. DNA extracts from the BT extraction kit were subjected to a magnetic bead purification protocol following extraction before qPCR analysis based on evidence of inhibition from BT kit extracts during initial qPCR tests. However, NW kit extracts may or may not have shown similar levels of inhibition. Therefore, whether purification may be necessary for NW kit extracts before qPCR analyses remains unknown. Due to biases introduced during DNA purification before qPCR and during metabarcoding library preparation, caution should be applied when comparing rates of detection success between analytical methods in the present study. Future studies may use a larger sample size and directly investigate levels of inhibition in samples before and after DNA extraction with each method to further validate these results across different taxa and environments, both in single-species and multi-species or community detection applications. Researchers seeking to compare detection success between qPCR, ddPCR, and metabarcoding in future studies should carefully consider the limitations imposed by methodological decisions, such as primer choice and pre-processing steps (*e.g*., inhibitor removal protocols), and the uncontrollable inherent biases of each analytical method.

## Supplemental Information

10.7717/peerj.20347/supp-1 Supplemental Information 1 Modified Qiagen DNeasy® Blood & Tissue kit DNA extraction method adapted for eDNA extraction from filters (Milián-García, 2022).

10.7717/peerj.20347/supp-2 Supplemental Information 2 ddPCR raw data. Raw data with dilution factor applied to facilitate comparison between BT and NW kits.

10.7717/peerj.20347/supp-3 Supplemental Information 3 MiFish raw data. Raw data with dilution factor applied to facilitate comparison between BT and NW kits.

10.7717/peerj.20347/supp-4 Supplemental Information 4 MetaWorks raw data. Raw data with dilution factor applied to facilitate comparison between BT and NW kits.

10.7717/peerj.20347/supp-5 Supplemental Information 5 qPCR raw data. Raw data with dilution factor applied to facilitate comparison between BT and NW kits.

10.7717/peerj.20347/supp-6 Supplemental Information 6 Qubit raw data. Raw data with dilution factor applied to facilitate comparison between BT and NW kits.

## Additional Information and Declarations

### DNA Deposition

The following information was supplied regarding the deposition of DNA sequences:

The raw data is available in the Borealis Research Data Repository:

Myler, Erika; Milian-Garcia, Yoamel; Loeza-Quintana, Tzitziki; Bourque, Danielle; Hanner, Robert H., 2024, “Optimization of environmental DNA-based methods: A case study for detecting brook trout (*Salvelinus fontinalis*)”, https://doi.org/10.5683/SP3/XOQMFB, Borealis, V1, UNF:6:S8hsVtOruW4zRX5SviuiDA== [fileUNF].

### Data Availability

The following information was supplied regarding data availability:

The raw data is available in the Borealis Research Data Repository:

Myler, Erika; Milian-Garcia, Yoamel; Loeza-Quintana, Tzitziki; Bourque, Danielle; Hanner, Robert H., 2024, “Optimization of environmental DNA-based methods: A case study for detecting brook trout (*Salvelinus fontinalis*)”, https://doi.org/10.5683/SP3/XOQMFB, Borealis, V1, UNF:6:S8hsVtOruW4zRX5SviuiDA== [fileUNF].
