# Supplementary material for: Optimization of environmental DNA-based methods: a case study for detecting brook trout (Salvelinus fontinalis)
Source: PeerJ. 2026 Feb 11;14:e20347. doi: 10.7717/peerj.20347 (PMC12906266; doi:10.7717/peerj.20347)
Supplement: Supplemental Information 1 [file peerj-14-20347-s001.docx]

Modified Qiagen DNeasy® Blood & Tissue kit DNA extraction method adapted for eDNA extraction from filters (Milián-García, 2022)

1. Cut the filter into quarters and place small pieces of each quarter in independent 2.0 ml microcentrifuge tubes with approximately 250 mg of glass beads. Add 380 μl Buffer ATL.
2. Disrupt every quarter before Proteinase K digestion using the Qiagen TissueLyser (1 min at 30 Hz). Spin the samples down.
3. Add 20 μl Proteinase K. Mix thoroughly by vortexing (10 sec), and incubate at 56°C (overnight), shaking at 700 r.p.m.
4. Vortex occasionally during incubation to disperse the sample or place it in a thermomixer, shaking water bath, or rocking platform. Lysis time varies depending on the type of sample processed. It is usually completed in 1–3 h. If it is more convenient, samples can be lysed overnight; this will not affect them adversely. Note that samples were lysed overnight for the current study.
5. Vortex for 15 s and spin down. Add 400 μl Buffer AL to the sample, and mix thoroughly by vortexing (10 sec). Incubate at 56°C (10 min).
6. Then add 400 μl ethanol (96–100%), and mix again thoroughly by vortexing (30 sec).
7. Pipet the mixture from step 5 (including any residue) into the DNeasy Mini spin column placed in a 2 ml collection tube (provided). Centrifuge at 11,000 x g for 1 min. Discard the flow-through and collection tube.
8. Place the DNeasy Mini spin column in a new 2 ml collection tube (provided), add 500 μl Buffer AW1, and centrifuge for 1 min at 11,000 x g. Discard flow-through and collection tube.
9. Place the DNeasy Mini spin column in a new 2 ml collection tube (provided), add 500 μl Buffer AW2, and centrifuge for 5 min at 20,000 x g to dry the DNeasy membrane. Discard flow-through and collection tube. Drying the DNeasy Mini spin column membrane is essential since residual ethanol may interfere with subsequent reactions. This centrifugation step ensures that no residual ethanol will be carried over during the following elution.
10. Following the centrifugation step, carefully remove the DNeasy Mini spin column so that it does not contact the flow-through. This will result in a carryover of ethanol. If a carryover of ethanol occurs, empty the collection tube and reuse it in another centrifugation for 1 min at 20,000 x g.
11. Place the DNeasy Mini spin column in a clean 1.5 ml or 2 ml microcentrifuge tube (not provided), and pipet 100 μl Buffer AE (prewarmed at 70 oC) directly onto the DNeasy membrane. Incubate at room temperature for 15 min, and then centrifuge for 5 min at 11000 x g to elute.
12. Elution with 100 μl (instead of 200 μl) increases the final DNA concentration in the eluate and decreases the overall DNA yield.
13. Pool the DNA extracts into a single tube.
